# DMS-SLAM: A General Visual SLAM System for Dynamic Scenes with Multiple Sensors

**DOI:** 10.3390/s19173714

**Published:** 2019-08-27

**Authors:** Guihua Liu, Weilin Zeng, Bo Feng, Feng Xu

**Affiliations:** School of Information Engineering, Southwest University of Science and Technology, Mian’yang 621010, China

**Keywords:** dynamic scenes, sliding window, Grid-based Motion Statistics (GMS), static 3D map points, accuracy and speed

## Abstract

Presently, although many impressed SLAM systems have achieved exceptional accuracy in a real environment, most of them are verified in the static environment. However, for mobile robots and autonomous driving, the dynamic objects in the scene can result in tracking failure or large deviation during pose estimation. In this paper, a general visual SLAM system for dynamic scenes with multiple sensors called DMS-SLAM is proposed. First, the combination of GMS and sliding window is used to achieve the initialization of the system, which can eliminate the influence of dynamic objects and construct a static initialization 3D map. Then, the corresponding 3D points of the current frame in the local map are obtained by reprojection. These points are combined with the constant speed model or reference frame model to achieve the position estimation of the current frame and the update of the 3D map points in the local map. Finally, the keyframes selected by the tracking module are combined with the GMS feature matching algorithm to add static 3D map points to the local map. DMS-SLAM implements pose tracking, closed-loop detection and relocalization based on static 3D map points of the local map and supports monocular, stereo and RGB-D visual sensors in dynamic scenes. Exhaustive evaluation in public TUM and KITTI datasets demonstrates that DMS-SLAM outperforms state-of-the-art visual SLAM systems in accuracy and speed in dynamic scenes.

## 1. Introduction

Simultaneous Localization and Mapping (SLAM) plays a major role in robot pose estimation, which is used in robot navigation [1,2], obstacle avoidance, VR (Virtual Reality) and AR (Augmented Reality) [3], etc. Due to its global optimization and closed-loop detection, the SLAM system can correct the accumulative error of the visual odometer (VO) during the tracking process when the robot revisits places. 

Since the camera can obtain rich scene information, the visual SLAM (VSLAM) for robot pose tracking through the camera has attracted extensive attention. The monocular camera is widely used in VSLAM owing to its small size and convenience. However, there are problems with the use of a monocular camera, e.g., scale ambiguity, complex initialization and weak system robustness. Therefore, we can obtain the absolute scale of the system by using stereo or RGB-D cameras, and the stability of the system can be enhanced at the same time.

**Figure 1:**
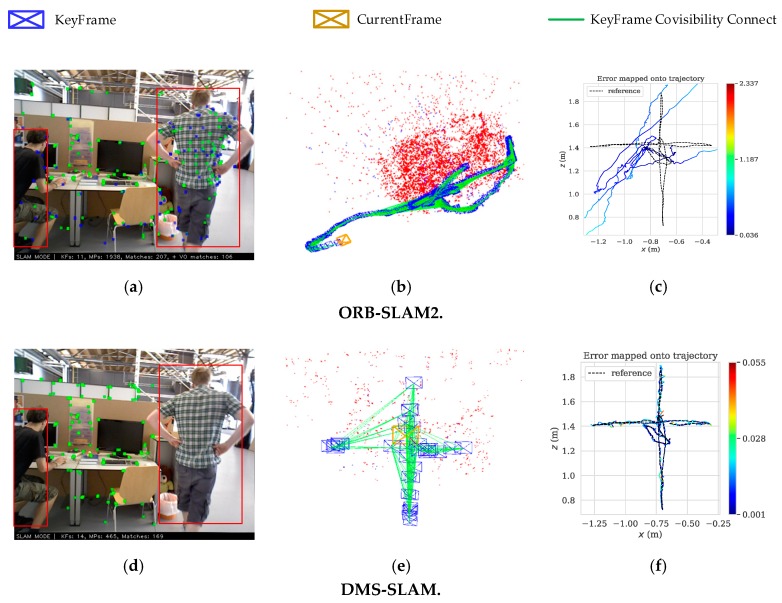
DMS-SLAM and ORB-SLAM2 use an RGB-D camera to locate and map on fr3_walking_xyz. (**a**,**d**) The image frame on fr3_walking_xyz. The red boxes represent moving objects. Green dots represent the ORB feature points corresponding to 3D points in the local map, while blue dots represent them only observed by the current frame. (**b**,**e**) The actual running trajectory that the camera takes during the move. The red represents the local map points which are observed in the current frame. The red and blue represent the local map points and are constructed during the tracking process. (**c**,**f**) The positioning deviation of the camera in the course of motion is obtained, which is based on the ground truth as the benchmark. Where “reference” represents the ground truth. The color represents the error value of the tracking trajectory and ground truth.

The above research has solved many problems of VSLAM. Many advanced SLAM algorithms have been proposed successively, such as in [4,5,6,7,8,9,10]. However, these algorithms are tested experimentally in static scenes or datasets. Although they can achieve good positioning accuracy and robustness, they do not address the issue of dynamic objects in scenes. The methods in [11,12] use the physical priors algorithm to achieve real-time monocular camera pose estimation in dynamic scenes, and both of them have excellent accuracy. In addition, they perform Structure from Motion (SfM) and real-time 3D reconstruction on the dynamic objects in the scene, and have high reconstruction accuracy. However, they all aim at non-rigid objects that move slowly in dynamic scenes, such as the face, the silicone cloth, the bending paper, etc. When there are fast moving rigid or non-rigid objects in dynamic scenes, e.g., people, cars, etc., VSLAM often have a large positioning error or cause tracking failure. Therefore, pose estimation and mapping in a dynamic environment is always a challenge. To solve the above problems, in this paper, we propose a novel algorithm for monocular, stereo and RGB-D initialization and pose tracking. In the initialization, the combination of the Grid-based Motion Statistics (GMS) [13] algorithm and the sliding window is used to get the static feature matching points for the initialization of VSLAM system. In the pose tracking part, only the keyframes are feature-matched to add 3D points in the local map. Then, the pose tracking is performed by 3D feature points reprojection. Our algorithm is built on GMS and ORB-SLAM2 [7], which are the most advanced SLAM and feature matching algorithms, respectively. Figure 1 represents an example of dynamic scene tracking and map building in TUM [14] dataset.

From Figure 1, since the original ORB-SLAM2 tracks the ORB feature points on the dynamic objects (Figure 1a). Therefore, there is a large deviation between the actual running trajectory (Figure 1b) and the ground truth of the camera, which is about 0.036 m–1.187 m (Figure 1c). However, our system eliminates the influence of feature points on dynamic objects and only tracks the feature points of static regions (Figure 1d), thus achieving stable tracking of camera pose (Figure 1e). The deviation between the actual trajectory and the ground truth of DMS-SLAM is within 0.001 m–0.028 m (Figure 1f), which has excellent positioning accuracy. The contributions of this paper can be seen as follows:

1. A novel method for VSLAM initialization is proposed to set up static initialization 3D map points.
2. Static 3D points are included in the local map through GMS feature matching algorithm between keyframes, and static global 3D map is constructed.
3. High accuracy and real-time monocular, stereo and RGB-D positioning and mapping system for dynamic scenarios.

The structure of the rest paper is as follows: introduction of related work in Section 2, overall description of the system in Section 3, detailed description of the method we proposed in Section 4, our experimental part in Section 5, summary and expectation the future work finally in Section 6.

## 2. Related Works

In this section, we review the existing visual SLAM algorithm, and how to eliminate the influence of dynamic objects in a dynamic scene to get the correct camera pose.

The earliest SLAM system uses the filtering [15,16,17] algorithm for pose estimation. The current frame and the previous frame are filtered to realize the initialization of the system and the pose tracking of the camera. However, the method is computationally intensive, and the pose of the current frame is only related to the previous frame. So the accumulative error in the pose tracking is large, and it is not suitable for long-term pose estimation. Therefore, a method based on keyframe and nonlinear optimization [18] is proposed. For example, SVO [4], ORB-SLAM [6], LSD-SLAM [5] and DSO [8], etc. These algorithms use a monocular camera for pose estimation, but the drawbacks of monocular camera are scale ambiguity and lack of robustness in tracking. Therefore, all the above algorithms are improved to adopt a stereo or RGB-D camera for pose tracking, such as in [7,19,20]. However, in the real environment of robot localization and mapping, if we only use the camera for pose estimation, tracking loss is likely to occur when the robot moves rapidly. Therefore, the vision is combined with an IMU (inertial measurement unit) sensor to improve the stability of the system, such as [21,22,23,24,25]. Although the above algorithms can achieve high positioning accuracy, most of them are verified in static scenes. In the dynamic scene, due to the existence of moving objects, which may cause tracking failure or large deviation of the SLAM system during pose estimation. At present, there are two core methods for eliminating the influence of dynamic objects: direct screening and deletion method and semantics segmentation method. The explanation of the two methods is as follows:

Direct Screening and Deletion: According to the keypoints of image frame extraction, such as Tomasi [26], FAST [27] and ORB [28], etc., the key points are filtered directly. The most representative is RANSAC [29] used in [3] and [6], which has a superior estimate for low dynamic scenes, but it still makes the estimation of position incorrectly or the tracking failure when there are moving cars or persons in the scene. According to the motion relationship between successive image frames, the optical flow method is used to dynamic object detection. For example, Fang et al. [30] combines optimal estimation and uniform sampling to perform dynamic object detection. Compared with the traditional optical flow method, this method is faster but less accurate. Wang et al. [31] used RGB optical flow method to segment dynamic objects in the scene, and Alcantarilla et al. [32] carried out dynamic object detection according to binocular stereo scene flow. However, they all need to provide prior information of dynamic objects, which is not suitable for long-term pose tracking.

Semantics Segmentation: Recently, deep learning is used to detect dynamic objects in the scene and segment them semantically. Based on ORB-SLAM2, Chao et al. [33] used SegNet [34] network for semantic segmentation to obtain semantic information in images, and combined with motion consistency check to remove feature points on dynamic objects. The algorithm constructs an octree map with semantic information, which has superior real-time performance (the running speed), but the less positioning accuracy in the tracking. Similarly, Bescos et al. [35] used Mask R-CNN [36] for image segmentation. For RGB-D cameras, dynamic feature points were detected and removed by combining multi-angle geometry. A static 3D dense map was realized by combining image Impainting technology. Although this algorithm has higher positioning accuracy, the system has lower real-time performance. Barsan et al. [37] used sparse scene stream and semantic segmentation for dynamic target recognition, and binocular stereo matching to construct static dense three-dimensional maps. However, the algorithm did not evaluate the positioning accuracy, and the running speed was only 2.5 Hz.

## 3. System Overview

**Figure 2:**
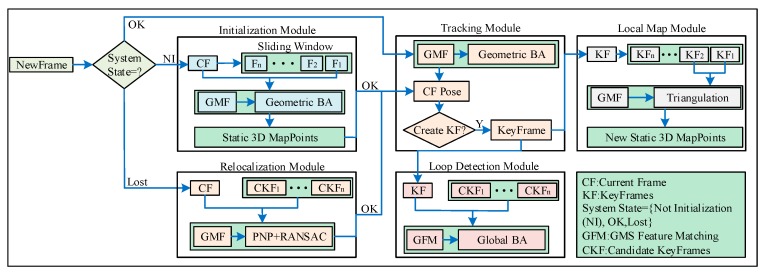
The system architecture diagram of DMS-SLAM. Our pipeline consists of initialization, tracking, local map, loop detection and relocalization module. System initialization is achieved according to NewFrame in the initialization module. The current frame pose estimation and keyframes are obtained in the tracking module after system is successfully initialized. In the local map module, a static local map is constructed according to key frames in the tracking module. When the system detects the closed-loop, it enters the loop detection module to optimize the global pose and map points and when the system tracking is lost, the relocalization module is entered to perform the current frame pose estimation.

We proposed a DMS-SLAM system which is based on the state-of-the-art ORB-SLAM2 [7]. It combines the latest GMS [13] feature matching algorithm (Section 4.1) to improve it. DMS-SLAM supports the construction of image frames of monocular, stereo and RGB-D visual sensors (Section 4.2). In addition, it can perform pose tracking on dynamic scenes and with the best positioning accuracy and speed. Figure 2 shows our system consists of five modules: initialization, tracking, local map, relocalization and closed-loop detection, and it starts to be uninitialized (System State = NI).

In the initialization module (Section 4.3), we use the sliding window to perform feature matching of all feature points in the dynamic scene, and then acquire static initialization 3D map points according to the BA (Bundle Adjustment) optimization algorithm.

When the system initialization is successful (System State = OK), it enters the tracking module (Section 4.4). The system achieves the pose estimation of the current frame and continuous image frame real-time positioning based on the static local map that was constructed.

The keyframes in the tracking module are sent to the local map module (Section 4.5). GMS feature matching is performed on two adjacent keyframes in the local map, and static feature matching between the two key frames is obtained. The number of map points in the local map is increased by triangulating the matched feature points into 3D points in the world coordinate system.

In the closed-loop detection module (Section 4.6), feature matching is performed by finding candidate keyframes that have covisibility with the current keyframe but are not directly connected. When the closed-loop is detected, the global BA algorithm is used to globally optimize the map points and keyframe’s poses.

If the current frame pose tracking is lost (System State = Lost), the system enters the relocalization module (Section 4.6). We use the GMS algorithm to perform feature matching between the current frame and the keyframe with the common view, and then determine whether the matching point has a corresponding 3D point in the local map. Making sure we get static feature matching. The current frame pose after relocalization is obtained by combining PNP [38] and RANSAC algorithm.

## 4. Methodology

In this section, the feature matching algorithm, image frame construction and various modules of the system are presented in detail.

### 4.1. Feature Matching

We use the GMS feature matching algorithm to enhance the original ORB-SLAM2 system, which is an efficient and real-time feature matching algorithm. The GMS algorithm uses BF (Brute Force) matching in OpenCV to achieve feature matching on two frames of images, and obtains the initial feature matching point pairs. Then, the original feature matching is filtered by the grid-based motion statistics model to obtain the correct feature matching.

**Figure 3:**
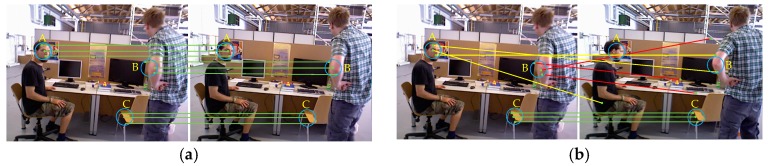
A and B represent partial motion areas in the scene, and C represents static areas in the scene. (**a**) Feature matching is performed using two consecutive image frames. Since only a small movement occurs in the dynamic region, the A, B and C regions can achieve excellent feature matching. (**b**) Feature matching between two consecutive image frames is achieved by the sliding window model. Due to the obvious movement of the dynamic region, the feature matching of region A and B is wrong, and the matching of region C is still correct.

In the feature point method SLAM system, two consecutive frames of image are generally used for feature matching to perform system initialization and pose tracking. However, when there are dynamic objects in the scene, we need to eliminate the feature matching point pairs on the dynamic object in order to achieve the correct feature matching. Because of the high frame rate of the camera, the moving object does not move significantly for two consecutive frames, so the influence of the dynamic object cannot be eliminated. Therefore, we use the sliding window model to achieve feature matching between the first frame and the nth (n indicates the size of the sliding window) frame image. In this way, the influence of the dynamic region is eliminated, and feature matching of the static region is obtained. Figure 3 shows the schematic diagram of feature matching between continuous image frames and discontinuous image frames.

According to the principle of GMS feature matching algorithm:

(1) $$S=|X_{i}|-1,$$

where S represents the score of a feature matching point pair in the region A, B or C, and $\left|X_{i}\right|$ represents the number of feature matching point pairs in the region A, B or C. If a feature matching point pair is matched correctly, there are still other feature matching point pairs around it, so the value of S is larger.

Two consecutive image frames are shown in Figure 3a. Since there is no dynamic object in the region C, the scores of the feature matching point pairs in the region are higher. However, the dynamic objects in the regions A and B do not significantly move between successive image frames, so there are still many feature matching point pairs in these area, and a larger S value is also obtained. Assume that the scores of the feature matching point pairs in the A, B, and C regions are $S_{A}$, $S_{B}$, and $S_{C}$ respectively; there is Formula (2):

(2) $$S_{A}\approx S_{B}\approx S_{C}$$

In this case, the feature matching of the dynamic regions (regions A and B) and the static region (region C) cannot be distinguished by setting an appropriate threshold. Therefore, the effects of dynamic objects in the scene cannot be eliminated.

As is shown in Figure 3b, if the sliding window is used, then we perform feature matching between two discontinuous image frames. There is no significant change in the static region C, and the scores of the feature matching point pairs are similar to the region C in Figure 3a. However, the areas A and B are affected by obvious movement of the dynamic objects, causing large errors in the feature matching of these regions. That led to small values of $S_{A}$ and $S_{B}$. Therefore, Formula (3):

(3) $$S_{A}\approx S_{B}\ll S_{C}$$

We can use the sliding window model to eliminate the effects of dynamic objects by setting the corresponding thresholds, thus obtain correct feature matching of static regions.

If the accuracy analysis is performed on each feature matching point pair in the image, the time complexity of the algorithm is higher. To ensure the speed of the algorithm, the image is meshed and the correctness of all feature matching point pairs in each grid is evaluated directly.

**Figure 4:**
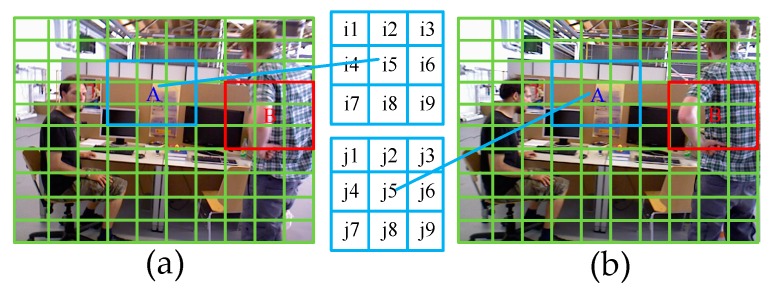
(**a**) Grid division of the first frame image. (**b**) Grid division of the Nth (the sliding window size) frame image. Where A and B represent the center area of a 3 × 3 grid corresponding to the left and right image.

Figure 4 shows the meshing of images. In the 3 × 3 grids centered on the grid A, the matching score of the right and left image centering area A is counted. The matching score $S_{ij}$ is defined as:

(4) $$S_{ij}=\sum_{k=1}^{K=9}\left|X_{i_{k}j_{k}}\right|$$

where $\left|X_{i_{k}j_{k}}\right|$ represents the number of matching points of the corresponding feature in the grid pair $\left\{i_{k},j_{k}\right\}$. 

According to the consistency of the smoothness of the motion and the matching of the features, the larger the matching score value, the greater the correctness of the feature matching in the grid A. Therefore, we set the threshold t to determine whether the feature matching in grid A is correct, which is given as:

(5) $$t=\alpha \sqrt{n}$$

where α is a constant (typically set to 6), *n* represents the number of all feature points in the 3 × 3 grids centered on A. For static areas (the blue boxes), the grid A can get a higher match score $S_{ij}$.

However, for the dynamic region (the red boxes in Figure 4), due to the existence of the moving object, the feature point extracted by the left image cannot find the corresponding matching point in the right image, or the wrong matching point in the right image, so the value of the matching score $S_{ij}$ is low. Therefore, we can set the adaptive threshold according to the number of feature points in the region, which cannot only eliminate the mismatch of the dynamic region, but also improve the matching effect of the static region.

### 4.2. Construct NewFrame

**Figure 5:**
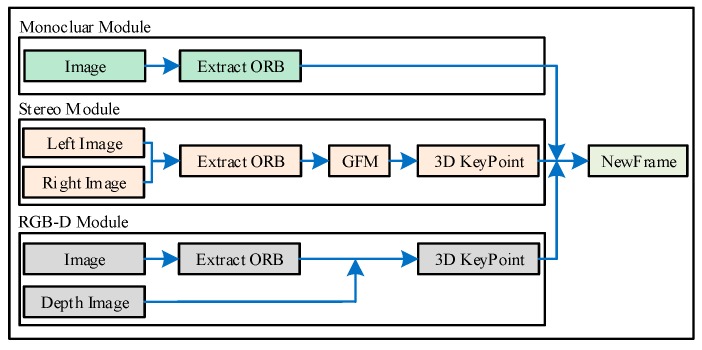
The pipeline shows image frame construction for monocular, stereo, RGB-D cameras. The GMS feature matching algorithm is mainly used in the stereo camera part to improve the matching speed and matching effect.

Before entering each module of the system, we need to build the current image information into a new image frame. The process of building image frames for our system is illustrated in Figure 5.

For monocular and RGB-D cameras, image frames are built in the same way as ORB-SLAM2. For stereo cameras, after extracting the uniform distribution of ORB features, the Grid-based Motion Statistics algorithm is used to match the ORB features of the left and right images. Then the parallax of the left and right matching points $\left(u_{L},v_{L}\right)$ and $\left(u_{R},v_{R}\right)$ is calculated. The depth value z of the matching feature points in the left image under the left camera coordinate system is obtained according to the Formula (6):

(6) $$z=\frac{f_{x}*b}{u_{L}-u_{R}}$$

where $f_{x}$ is the camera internal parameter and b is the baseline distance of the stereo camera.

Compared with the epipolar constraint algorithm in ORB-SLAM2, we use the method with faster matching speed, which can improve the speed of image frame construction of stereo vision SLAM system (we will illustrate this in Section 5).

### 4.3. System Intialization

We use the sliding window to achieve GMS feature matching of two frames of images, and obtain a relatively static initialization 3D map, as is shown in the initialization module in Figure 2. Since the positional transformation of moving objects in the image is not obvious in successive image frames, we create a sliding window to achieve feature matching between multiple image frames. We save n image frames in the sliding window (n = 7, which is experimentally determined). When the new image frame is established, it enters the initialization module if the system is not initialized. The current image frame and the first image frame (F1 in Figure 2) in the sliding window are matched by the GMS algorithm. In this way, the influence of moving objects in the scene is eliminated, and feature matching of the static region is obtained.

**Figure 6:**
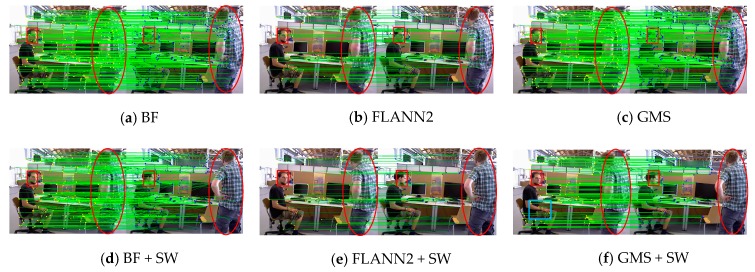
SW represents the sliding window, and the red box/ellipse indicates that the object in the area has moved. (**a**–**c**) Three matching algorithms use two consecutive frames of images to achieve feature matching on the fr3_walking_xyz dataset in TUM. (**d**–**f**) These algorithms combine the sliding window model to perform feature matching between the first frame image and the nth frame image. GMS with SW can get the best feature matching result and eliminate the influence of moving objects in the red area.

Figure 6 shows that we use three feature matching algorithms, including classical BF, regionalized nearest neighbor (referred to as FLANN2) in ORB-SLAM2 and GMS, to match the ORB feature points of the image pair (in the BF algorithm, we use the classic Hamming distance to filter the matching feature points, setting a smaller distance threshold of 0.6). As is illustrated in Table 1, in feature matching, we set the extraction of 1000 ORB feature points, and count the matching time of the three algorithms and the number of matching feature points.

According to Table 1 and Figure 6, BF feature matching is the fastest matching algorithm. The main reason is that we only use the most basic distance threshold to filter it, so it can achieve a high speed. However, BF matching algorithm still cannot eliminate the influence of dynamic objects after using the sliding window model, and there are many mismatches (Figure 6a,d). For the FLANN2 feature matching algorithm, the impact of dynamic objects can be eliminated by combining the sliding window model (Figure 6b,e). However, feature matching of this algorithm takes a long time and the number of feature matches obtained is fewer, so there will be initialization failure or tracking loss in the tracking process. We use the combination of GMS and sliding window to achieve feature matching in the system initialization module, which not only has an efficient matching speed, but also eliminates the influence of dynamic objects in the scene, and obtains a rich static feature matching point pair (Figure 6c,f).

Please note that in the blue box of Figure 6f, since this area is not moved in the current scene, feature matching can be performed on this area to build local 3D map points. Although the feature matching obtained in this area is relatively static in the current scene, the region is a potential motion area. During the tracking process, if motion occurs in the area, the map point is filtered through BA optimization and the proportion of image frames that can observe the 3D point of the region. If there are relatively few moving objects in the scene, our method also has obvious advantages in the matching speed and number of feature points.

After getting the correct feature matching points, we use them to initialize the system. For the monocular camera, the score *S* of the homography matrix H and the basic matrix F is calculated by the reprojection error based on the obtained feature matching points, and the threshold *T* is obtained, which is shown in Formula (7):

(7) $$T=\frac{S_{H}}{S_{H}+S_{F}}$$

If *T* > 0.45, which is determined by experiment, the plane structure is used to initialize. Otherwise, we initialize the system according to the epipolar constraint, and finally the optimization of map points is initialized according to geometric BA optimization.

For stereo cameras, we get the 3D coordinates of static matching points directly according to the depth information of feature points obtained in image frame construction. For the RGB-D camera, based on the acquired disparity map, the 3D coordinates of the matching points are restored, and the initialization map points are constructed. Finally, 3D map points are optimized by BA.

**Figure 7:**
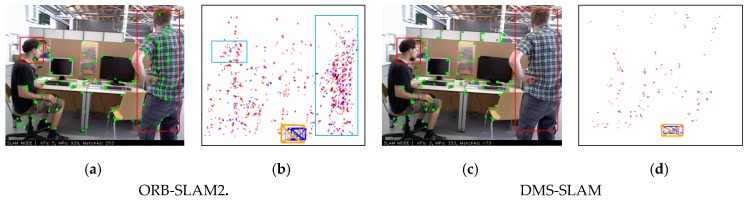
(**a**,**c**) System initialization results of ORB-SLAM2 and DMS-SLAM on the fr3_walking_xyz dataset of TUM, and the red boxes indicate the area that is moving. (**b**,**d**) The local map is built after system initialization, and the blue boxes represent the dynamic 3D points in the local map.

Figure 7 shows the result of ORB-SLAM2 and DMS-SLAM using RGB-D cameras for initialization. ORB-SLAM2 adds feature points on the dynamic object to the local map during the initialization process. However, our method can construct a relatively static 3D map during the initialization process. We highlight that the initialization map we construct consists of relatively static 3D map points. When the scene changes from static to motion, we will filter the original map points to build a static 3D map.

### 4.4. Tracking

After the initialization is successful (System State = OK), the pose information of the current frame is obtained and the first initialized frame is set as the keyframe and the reference frame. When the new image frame is built and goes directly to the tracking module, we use the reference frame model or the constant velocity model to estimate the current frame pose.

For the reference frame model, we set the latest keyframe as the reference frame, and GMS feature matching is carried out for the reference frame and the current frame. If the feature points matched by the reference frame have corresponding 3D points in the local map, then these 3D points are projected to the current frame. The pose information from the previous frame is used as the initial value of the current frame pose. Then, the pose information of the current frame and the observed 3D map points are acquired according to the BA optimization. In contrast to the reference frame model, the constant velocity model assumes that the motion between two frames is the same as the previous two frames, so as to set the initial value of BA optimization pose.

During the tracking process, keyframe selection is required to preserve key information during the tracking process. Unlike ORB-SLAM2, the interval between two keyframes requires more than 8 frames which is experimentally determined. This is primarily to exclude the effects of dynamic objects in the scene when adding new map points to the local map.

### 4.5. Local Mapping

The keyframes obtained in the tracking module are inserted into the local map module, and the keyframes are processed when more than two keyframes exist in the local map.

**Figure 8:**
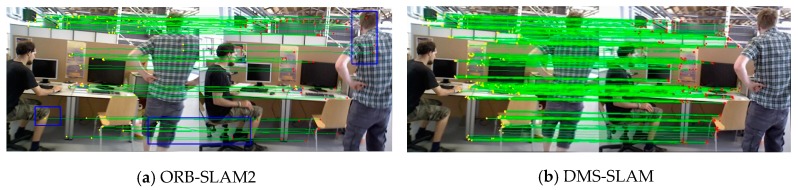
(**a**,**b**) Feature matching results between keyframes in ORB-SLAM2 and DMS-SLAM. The blue boxes in the figure indicate the dynamic area or the wrong feature matching point pairs.

For a new keyframe, the keyframe is matched with the previous keyframe by GMS feature, and then the feature matching point pair is triangulated to obtain its 3D coordinates under the world coordinate system. Figure 8 shows the keyframe matching results of ORB-SLAM2 and DMS-SLAM. Our approach not only eliminates the impact of dynamic scene, obtaining the feature matching of static areas, but also has more feature matching point pairs than ORB-SLAM2, and can add enough 3D points for local map to ensure the stability of tracking.

Before adding a new map point to the local map, we need to filter the previous map points. Assume that the number of occurrences of a feature point in the field of view is V, the number of times of tracking this feature is T, and the threshold is set to 0.5 in the experiment. Define the tracking score S=T/V. If S<0.5, the corresponding 3D map points will be rejected. 

**Figure 9:**
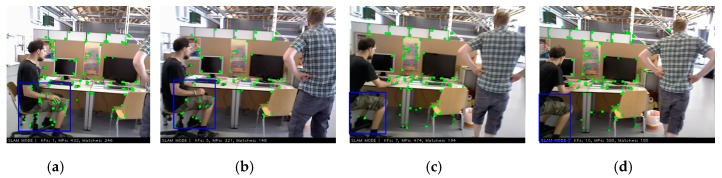
The order is (**a**), (**b**), (**c**), (**d**). They indicate the tracking process of our system. Where the blue box indicates that the area changes from static to motion, deletes the map point corresponding to the region’s feature point.

Figure 9 shows that DMS-SLAM removes the 3D map points corresponding to the feature points of the area when the original stationary area turns into a moving state. Compared to ORB-SLAM2 in Figure 1, we ensure that static map points are constructed in the local map.

After the keyframes in the local map have been processed, which are obtained from the tracking module. If the closed-loop detection module is not executed, the keyframes poses and the map points in the local map are optimized for local BA algorithm. In addition, if the map point repetition rate between two keyframes reaches 90% or more, the next keyframe will be deleted to avoid information redundancy between keyframes.

### 4.6. Relocalization and Loop Detection

When there are more moving objects in the scene, the static feature points are insufficient, or the camera moves too fast, the pose tracking is lost, so that the relocalization module is entered. Similar to the ORB-SLAM2, we calculate candidate keyframes that have covisibility with the current frame through DBoW2 [39]. Then, we match the current frame with the candidate keyframes sequentially using the GMS feature matching algorithm. If the matching feature points are greater than 30, the PNP and RANSAC algorithms are combined to estimate the pose of the current frame, and the BA optimization algorithm is used to obtain the corresponding 3D points of the current frame in the local map. If the number of 3D points of the current frame is recovered greater than 50, the relocalization succeeds and the tracking module is entered.

During the tracking process, when moving to the same position, the system needs to perform closed-loop detection, and global pose optimization is achieved to eliminate the accumulative error. For a new keyframe, we also use DBoW2 to calculate covisibility with the all keyframes and take keyframes that have covisibility but not directly connected as candidate keyframes. We perform feature matching between candidate keyframes and current keyframe by GMS algorithm, and combine PNP and RANSAC algorithms to solve and optimize poses (7-DOF (degrees-of-freedom) for monocular cameras and 6-DOF for stereo and RGB-D cameras). After determining the closed-loop keyframes, the pose graph is used to optimize the pose of all keyframes in the local map. Finally, the BA algorithm is used to optimize all keyframes and map points in the entire local map.

## 5. Evaluation

We experimented with RGB-D and monocular cameras on the TUM dataset and tested with stereo cameras on the KITTI [40] dataset. Meanwhile, with the most advanced SLAM system, the accuracy and speed are compared on two public datasets (including datasets of high dynamic scenes and low dynamic scenes). It is noted that:

1. In this paper, our RGB-D and monocular camera experiments were performed on the fr3 series of datasets with ground truth in TUM. s refers to sitting, w to walking and hs to halfsphere.
2. The available information is reduced due to the elimination of the effects of dynamic objects in the scene. Therefore, in the following experiments, we extract 1500 ORB feature points for analysis and comparison of accuracy and speed.
3. In the TUM dataset, the camera’s frame rate is 30, and the sliding window size is set to 7 determined by the experiment. However, in the KITTI dataset, the camera’s frame rate is 10, so setting the sliding window size to 3 to eliminate the effects of dynamic objects.
4. In contrast to the semantic segmentation method which is used to eliminate the effects of dynamic objects through GPU acceleration, e.g., DynaSLAM, DS-SLAM, all our experimental results are only run on the CPU (Intel i5 2.2 GHz 8 GB).

### 5.1. RGB-D

We use low dynamic scenes (similar to a static scene) and high dynamic scenes (there are moving objects in the scene) in the TUM dataset for experimental testing, and use RMSE (Root Mean Squared Error) of ATE (Absolute Trajectory Error) [14] to achieve localization accuracy evaluation. We also make statistics on the tracking time of the system to determine real-time performance of the SLAM system. Meanwhile, the state-of-the-art visual SLAM algorithms, i.e., DynaSLAM [35], DS-SLAM [33], ORB-SLAM2 [7], which are used for comparative analysis of localization accuracy and real-time performance (it has the same meaning as the system running speed).

Table 2 shows the accuracy and speed of DMS-SLAM and other visual SLAM in low and high dynamic scenes using RGB-D cameras. Please note that we use the tracking time (the time required to construct a single image frame to get the current frame pose) for speed evaluation. Because the RGB-D camera can get depth information and initialize the system directly, so we compare and analyze the average of the statistical tracking time.

For positioning accuracy, in low dynamic scenes, although our system and other SLAM systems have high positioning accuracy, our system has the highest positioning accuracy in most datasets compared to DynaSLAM and DS-SLAM systems. In addition, our system is built on ORB-SLAM2 and uses a better feature matching algorithm than ORB-SLAM2. Therefore, the positioning accuracy is improved compared to the ORB-SLAM2 system. In the highly dynamic scenes, compared with the ORB-SLAM2 system, our system avoids the error of pose estimation due to the influence of dynamic objects, and the accuracy is much higher than ORB-SLAM2 system. Moreover, on all datasets (w_*), our system has the highest positioning accuracy compared to DynaSLAM and DS-SLAM systems.

For running speed, our system is faster than the original ORB-SLAM2 because it removes the feature points on the dynamic objects in the scene during the tracking process and builds a static local 3D map. DynaSLAM and DS-SLAM use deep learning for semantic segmentation to eliminate the effects of dynamic objects in the scene. Although GPU acceleration is used in the semantic segmentation process, it still does not achieve superior real-time performance, especially for DynaSLAM. Combined with running speed and positioning accuracy analysis, our system not only has high positioning accuracy in dynamic scenes, but also has superior real-time performance, and the overall effect outperforms other SLAM systems.

**Figure 10:**
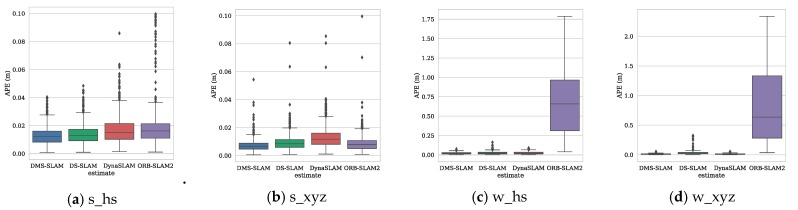
APE distribution of several visual SLAM algorithms obtained in TUM by using low dynamic (s_*) and high dynamic (w_*) datasets. The rectangular portions represent the distribution area of 3/4 APE data, and the other portions represent the distribution area of the remaining APE data. The top of the graph (horizontal line or small black dot) indicates the maximum value of APE and the bottom line represents the minimum.

To further illustrate the positioning accuracy of the system, we use the evo (https://github.com/MichaelGrupp/evo) tool to draw the absolute pose error (APE) and the motion trajectory. Figure 10 shows the positioning error of several visual SLAM systems during the tracking process on the four datasets s_hs, s_xyz, w_hs and w_xyz.

**Figure 11:**
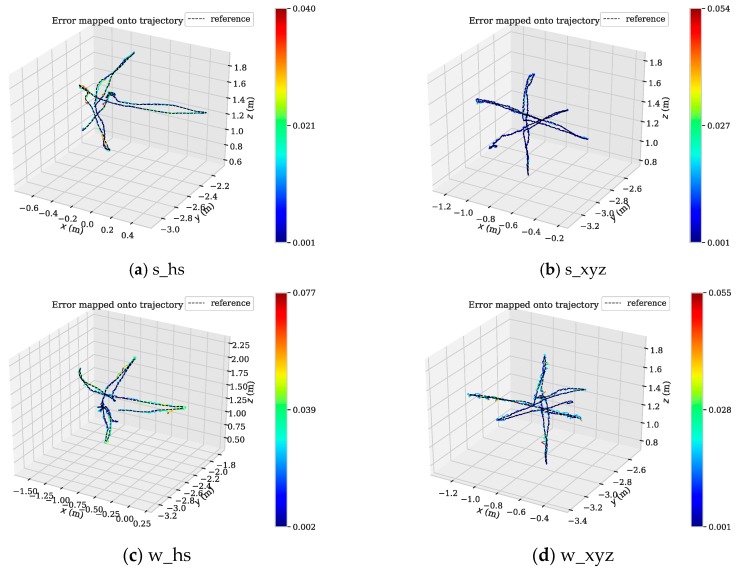
(**a**–**d**) The motion trajectory of DMS-SLAM in low dynamic (s_*) and high dynamic (w_*) datasets, where “reference” represents the Ground Truth. Where x and z represent the horizontal direction, and y represents the vertical horizontal direction.

From Figure 10, we can see that the APE of several SLAM algorithms in the positioning process of low dynamic datasets (s_*) is little, and the DMS-SLAM system has the highest positioning accuracy. In the highly dynamic datasets (w_*), the original ORB-SLAM2 has a large positioning error due to the influence of dynamic objects. We eliminate the effects of dynamic objects, so we can reach the highest positioning accuracy, and have better real-time performance than DS-SLAM and DynaSLAM. Figure 11 shows the motion trajectory of our method with reference to the ground truth in the above four datasets. It can be noted that DMS-SLAM can achieve accurate estimation of camera pose in dynamic scenes.

### 5.2. Monocular

For monocular camera, the dynamic scene datasets in TUM are also used for positioning accuracy and running speed testing. It should be noted that since the scale uncertainty for monocular cameras, the Lie group-Lie algebra Sim (3) is used for trajectory alignment to calculate the positioning accuracy. In addition, similar to DynaSLAM, we added the completeness rate of the track (*TCR*) as one of the evaluation criteria. Assume that the total number of image sequences in a dataset is *M*, and the number of image frames successfully tracked after the monocular initialization is *F*, then the *TCR* is defined as:

(8) TCR=F/M×100%

Table 3 shows the positioning accuracy, speed and *TCR* of DMS-SLAM and other visual SLAM systems in dynamic scenes. For running speed, since the monocular vision SLAM needs to be initialized from the motion structure, the feature points extracted for initialization are twice the default value. Therefore, in order to evaluate the tracking time more accurately, we use the median for comparative analysis. *TCR* means that if there are more image frames used for system initialization or tracking loss, the smaller the value of *TCR*. This indicator can therefore be used to measure whether the system can be quickly initialized and achieve stable pose tracking.

As is shown in Table 3, for positioning accuracy, although the ORB-SLAM2 system has higher positioning accuracy in dynamic scenes (this is due to the use of the RANSAC algorithm for monocular initialization, adding map points to local maps by FLANN2 feature matching between keyframes), the system initializes with difficulty due to the influence of moving objects, so the *TCR* value is significantly lower than DMS-SLAM (e.g., s_rpy, s_static, w_hs, w_xyz). Moreover, our system has the highest positioning accuracy in most datasets, because the GMS algorithm provides excellent feature matching point pairs. Although positioning accuracy in the s_static and s_rpy datasets is lower than DynaSLAM and ORB-SLAM2, its *TCR* value is much lower than our system.

For running speed, although both DMS-SLAM and ORB-SLAM2 have superior real-time performance, on most datasets, our system is faster than ORB-SLAM2. Since, DynaSLAM is affected by the deep learning module, its real-time performance is much lower than our system. It is a non-real-time SLAM algorithm.

**Figure 12:**
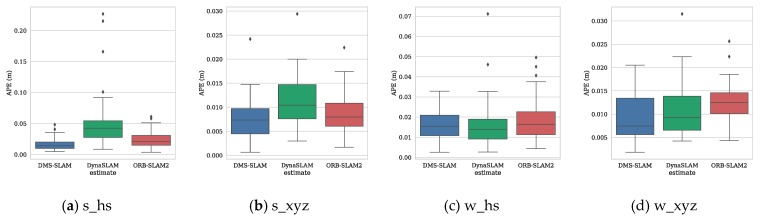
(**a**–**d**) We use Sim (3) for trajectory alignment and plot the APE distribution of the three monocular vision SLAM systems in low dynamic (s_*) and high dynamic (w_*) datasets.

**Figure 13:**
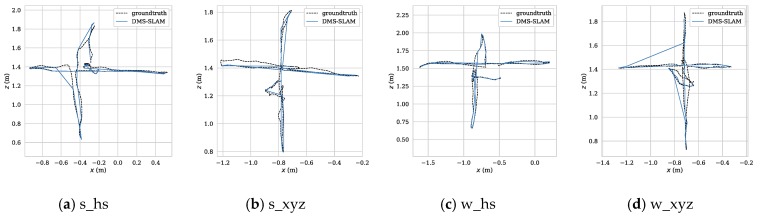
(**a**–**d**) We use Sim (3) for trajectory alignment and plot pose tracking graphs in low dynamic (s_*) and high dynamic (w_*) scenes of the DMS-SLAM system use monocular camera. Where x and z represent the horizontal direction.

Similarly, we use the evo tool to achieve APE and motion trajectory plotting for monocular vision SLAM systems in low dynamic (s_*) and high dynamic (w_*) scenes. Figure 12 shows the distribution range of APE on the four datasets of s_hs, s_xyz, w_hs and w_xyz for the three SLAM systems. From the figure, our system gets the best accuracy. Figure 13 shows the ATE of the DMS keyframes during tracking, and our system can achieve stable pose tracking in low dynamic and high dynamic scenes.

### 5.3. Stereo

**Figure 14:**
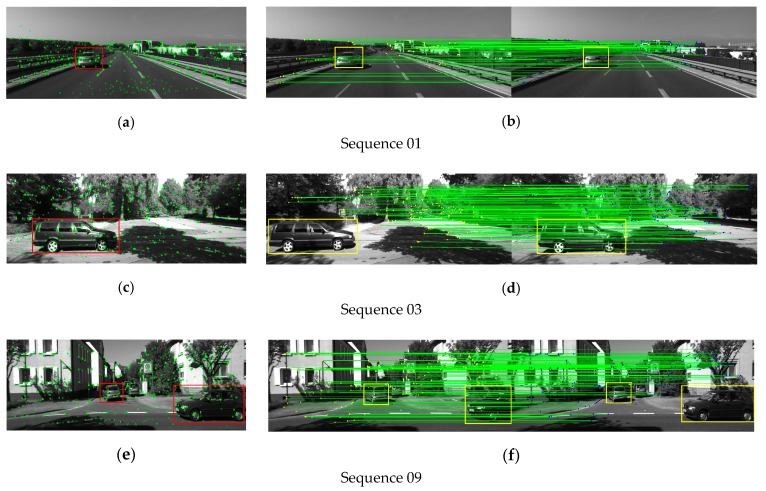
The DMS-SLAM extracts (**a**,**c**,**e**) and matches (**b**,**d**,**f**) experimental results of the ORB feature points on the 01, 03, 09 sequence in the KITTI datasets. The rectangular box represents the moving object in the scene, and the others are static areas.

The KITTI dataset is composed of urban and highway images. It gets the characteristics of long distance, variable environment and dynamic objects. For visual SLAM, there are enormous challenges. In the previous experimental analysis, we used the combination of the sliding window and GMS to achieve feature matching between two image frames in the TUM datasets. To ensure the integrity of the experiments in the article, we also implement the experiments of feature matching and pose tracking in the KITTI datasets. Since we have explained the necessity of combining sliding windows with GMS algorithms, therefore, the results of the feature matching experiments are directly obtained according to our algorithm, and are no longer compared with other algorithms. Figure 14 shows the feature extraction and matching results of the 01, 03, 09 sequences in the KITTI datasets.

**Figure 15:**
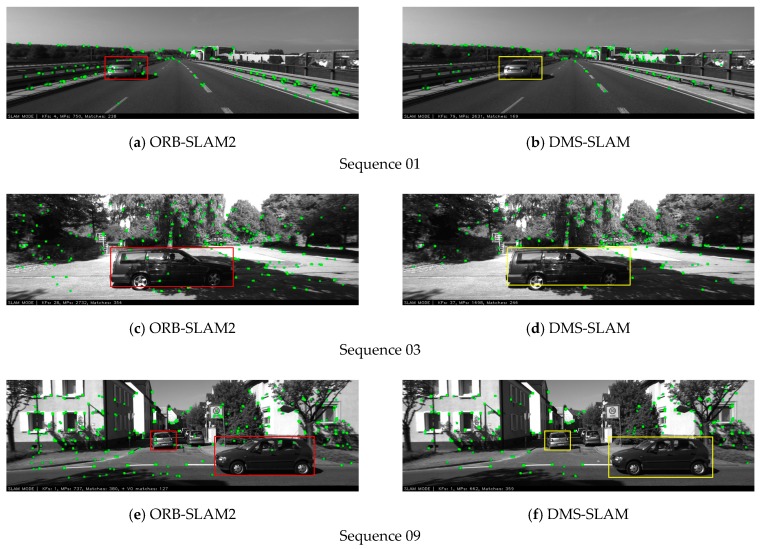
The pose tracking experiments of ORB-SLAM2 and DMS-SLAM on the 01, 03, 09 sequences in the KITTI dataset. The rectangular box represents the moving object in the scene, and the others are static areas.

Our algorithm can also eliminate the influence of dynamic objects on the KITTI datasets and obtain the feature matching points of the static regions in the scene. Figure 15 shows the local map points tracked by our algorithm and the original ORB-SLAM2 algorithm during the pose estimation. Our algorithm does not track feature points on dynamic objects during camera pose estimation, so influence of dynamic objects on pose estimation is eliminated.

After evaluating the feature extraction and matching of our algorithm, we implement the accuracy and real-time analysis of the stereo camera on the KITTI dataset. We use the first 11 sequences with ground truth for experimental testing, and Table 4 shows the experimental results of several stereo vision SLAM algorithms.

In terms of positioning accuracy, our algorithm eliminates the influence of dynamic objects in the scene and obtains better feature matching, so it has the highest positioning accuracy in most datasets. The reason the positioning accuracy in some datasets is slightly lower than other algorithms is that although we have excellent matching of the feature points in the two frames, the true depth value of the matched feature points is too large, which makes the depth estimation have large errors.

For running speed, we replace the original epipolar constraint with GMS feature matching, which speeds up the binocular stereo matching process and improves the speed of the original ORB-SLAM2 algorithm. Since DynaSLAM is a non-real-time SLAM system, we no longer count its speed.

**Figure 16:**
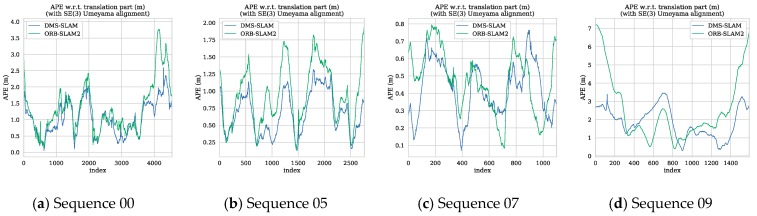
(**a**–**d**) In the sequence of KITTI dataset 00, 05, 07, 09, we use SE (3) to align the motion trajectory with the Ground Truth, and obtain the APE distribution of ORB-SLAM2 and DMS-SLAM.

**Figure 17:**
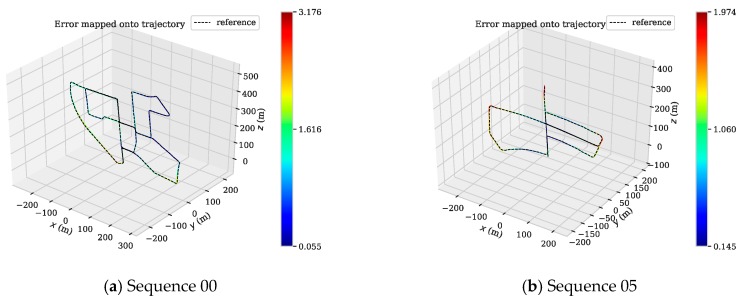

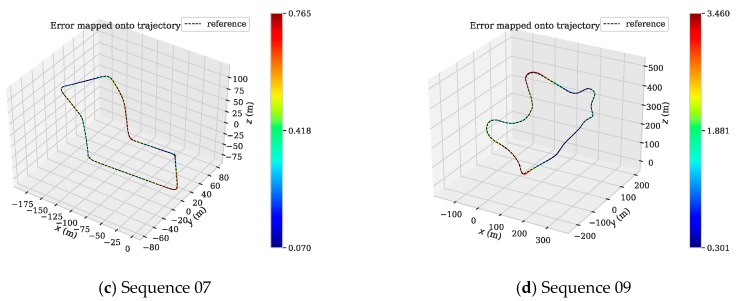
(**a**–**d**) Motion trajectory of DMS-SLAM on the 00, 05, 07, 09 sequences in the KITTI dataset. Where x and z represents the horizontal direction, and y represents the vertical horizontal direction.

We also use the evo tool to plot the APE and trajectory of the 00, 05, 07, 09 sequences in the KITTI dataset (Figure 16 and Figure 17). Since DynaSLAM is a non-real-time SLAM system, we no longer draw its APE and use only the data from the original paper for comparative analysis. Figure 16 shows the APE distribution of the DMS-SLAM and ORB-SLAM2 systems throughout the tracking.

As can be seen from Figure 16, our system’s accuracy in these sequences is significantly better than the original ORB-SLAM2 system. Figure 17 is the tracking trajectory obtained in the same sequence and the DMS-SLAM is consistent with the ground truth during the whole tracking and has high accuracy.

Since the KITTI dataset is a large-scale urban or highway environment, there is an accumulative error between two frames during the tracking, so its accuracy depends on the global BA optimization after detecting the closed loop. However, in the localization and mapping of the actual environment, especially for large-scale scenes, the car often do not go through the same scene, so closed-loop information is often difficult to obtain. This is a major challenge for current SLAM systems without closed-loop information to eliminate accumulating drift. Similar to VINS-Fusion [41], we can optimize the entire tracking trajectory by GPS to improve the accuracy of the SLAM system.

## 6. Conclusions

To solve the problem that the visual SLAM system generates a large deviation in the pose estimation, due to the existence of the moving object in the dynamic scene, we propose a visual SLAM system for monocular, stereo, and RGB-D cameras for dynamic scenes. The system is built on the ORB-SLAM2, and it is initialized by combining the GMS feature matching algorithm and the sliding window model to construct a static initialization 3D map. Then, according to the GMS feature matching algorithm and the map point selection method between keyframes, static map points are added to the local map to create a static 3D map that can be used for pose tracking. For RGB-D cameras, our system not only has extremely high positioning accuracy, but also has excellent real-time performance in dynamic scenes compared to other SLAM systems. In the monocular SLAM algorithm, our method is capable of long-term pose estimation and has high positioning accuracy. In stereo camera testing, we improved the speed of the original ORB-SLAM2 system, and our method has the best positioning accuracy in most datasets compared to other SLAM algorithms.

However, when the camera moves too fast, the system cannot track the feature points in time, or the system is located a low-texture scene. In these cases, the camera pose tracking will fail. Therefore, in future work, we can increase the stability of the system by adding IMU sensors and using multi-sensor fusion to achieve pose tracking.

## Figures and Tables

**Table 1:** Feature point matching time and quantity.

Methods	No Sliding Window		With Sliding Window	
	Times (ms)	Matches	Times (ms)	Matches
BF	5.23	627	5.40	401
FLANN2	20.93	124	13.21	65
GMS	6.01	650	6.27	318

**Table 2:** Accuracy and speed in low dynamic scenes (s_*) and high dynamic scenes (w_*).

Datasets	ATE RMSE (cm)				Tracking Time (s)			
	DMS-SLAM	Dyna SLAM	DS-SLAM	ORB-SLAM2	DMS-SLAM	Dyna SLAM	DS-SLAM	ORB-SLAM2
s_hs	1.406	1.913	1.481	2.307	0.051	1.242	0.172	0.098
s_rpy	1.757	3.017	1.868	1.775	0.046	1.565	0.124	0.077
s_static	0.687	0.636	0.610	0.960	0.041	1.294	0.142	0.079
s_xyz	0.800	1.354	0.979	0.817	0.052	1.557	0.135	0.078
w_hs	2.270	2.674	2.821	35.176	0.047	1.244	0.152	0.092
w_ rpy	3.875	4.020	37.370	66.203	0.048	1.273	0.146	0.081
w_ static	0.746	0.800	0.810	44.131	0.045	1.134	0.115	0.072
w_ xyz	1.283	1.574	2.407	45.972	0.046	1.184	0.136	0.079

**Table 3:** Accuracy, speed and *TCR* in low dynamic scenes (s_*) and high dynamic scenes (w_*).

Datasets	ATE RMSE (cm)			Tracking Time (s)			*TCR* (%)		
	DMS-SLAM	Dyna SLAM	ORB-SLAM2	DMS-SLAM	Dyna SLAM	ORB-SLAM2	DMS-SLAM	Dyna SLAM	ORB-SLAM2
s_hs	1.729	1.964	1.954	0.052	0.763	0.055	99.19	94.68	98.92
s_rpy	1.661	2.160	1.251	0.046	0.820	0.046	91.83	54.39	73.66
s_static	0.621	0.379	0.882	0.040	0.770	0.043	98.02	41.30	48.23
s_xyz	0.928	1.058	0.974	0.054	0.810	0.053	95.64	94.77	95.96
w_hs	1.859	1.965	1.931	0.047	0.754	0.049	97.75	97.00	89.22
w_ rpy	4.086	5.023	6.593	0.041	0.792	0.041	89.45	84.29	88.68
w_ static	0.204	0.342	0.498	0.039	0.761	0.039	86.81	84.79	86.54
w_ xyz	1.090	1.370	1.368	0.044	0.807	0.049	99.07	97.02	88.24

**Table 4:** Positioning accuracy and tracking time of stereo vision SLAM on KITTI datasets.

Datasets	ATE RMSE (m)			Tracking Time (s)	
	DMS-SLAM	DynaSLAM	ORB-SLAM2	DMS-SLAM	ORB-SLAM2
KITTI00	1.1	1.4	1.3	0.082	0.132
KITTI01	9.7	9.4	10.4	0.081	0.141
KITTI02	6.0	6.7	5.7	0.080	0.125
KITTI03	0.5	0.6	0.6	0.080	0.135
KITTI04	0.2	0.2	0.2	0.077	0.132
KITTI05	0.7	0.8	0.8	0.083	0.130
KITTI06	0.6	0.8	0.8	0.082	0.140
KITTI07	0.4	0.5	0.5	0.080	0.128
KITTI08	3.2	3.5	3.6	0.084	0.127
KITTI09	2.1	1.6	3.2	0.079	0.133
KITTI10	1.0	1.2	1.0	0.081	0.121
